# Supplementary material for: The interactome of intact mitochondria by cross-linking mass spectrometry provides evidence for coexisting respiratory supercomplexes
Source: Mol Cell Proteomics. 2017 Dec 8;17(2):216–32. doi: 10.1074/mcp.RA117.000470 (PMC5795388; doi:10.1074/mcp.RA117.000470)
Supplement: Supplemental Data [file supp_RA117.000470_133922_0_supp_23978_fzffwf.zip › spectra_annotation/mito_spectra_annotation/364-1-4-1-8-1.pdf]

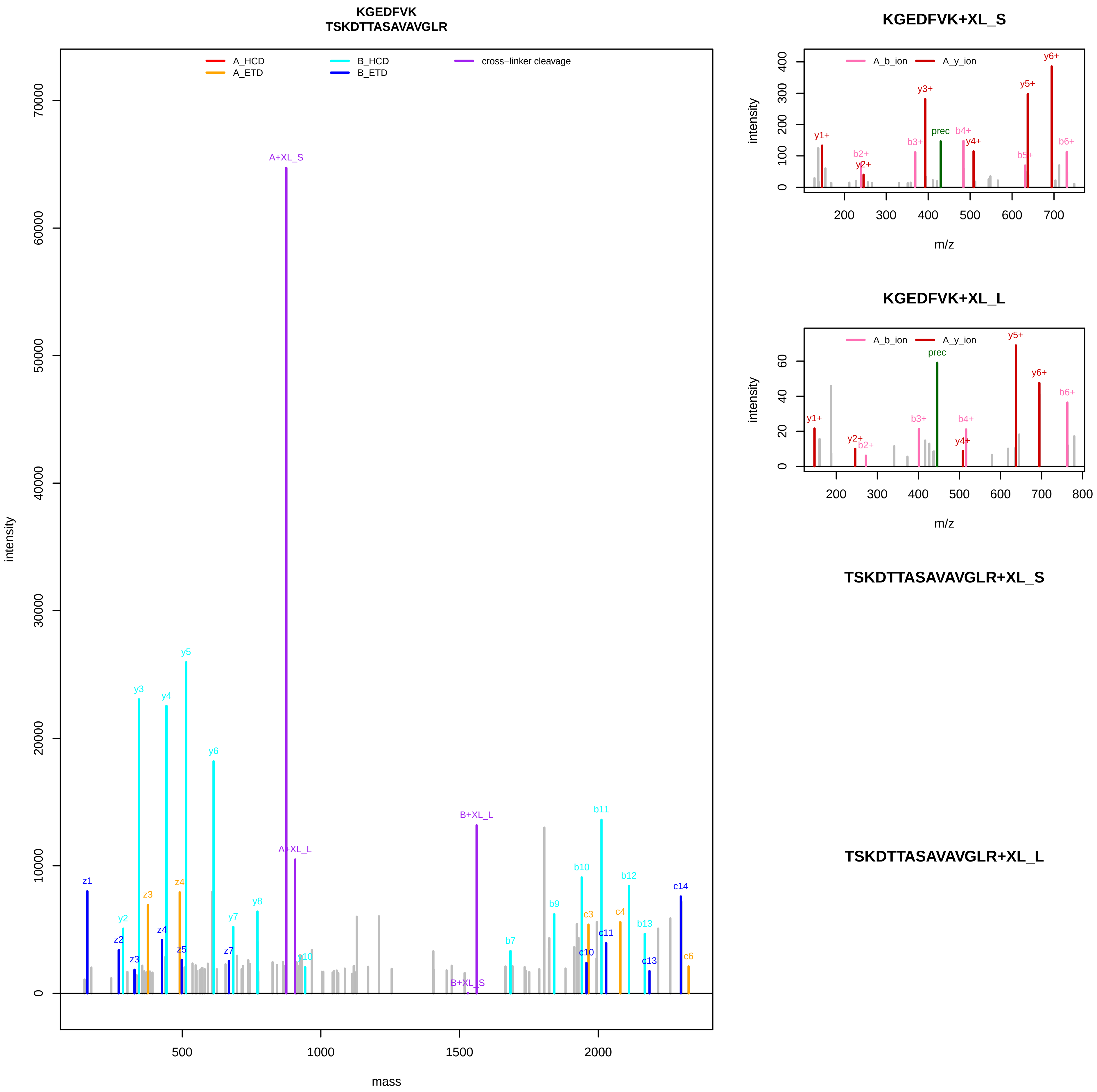

KGEDFVK
TSKDTTASAVAVGLR
A_HCD
A_ETD
B_HCD
B_ETD
cross−linker cleavage
intensity
mass
0
10000
20000
30000
40000
50000
60000
70000
500
1000
1500
2000
A+XL_S
A+XL_L
B+XL_L
B+XL_S
z1
z2
z3
z4
z5
z7
y2
y3
y4
y5
y6
y7
y8
y10
z3
z4
b7
b9
b10
b11
b12
b13
c3
c4
c6
c10
c11
c13
c14
KGEDFVK+XL_S
A_b_ion
A_y_ion
intensity
m/z
0
100
200
300
400
200
300
400
500
600
700
y1+
b2+
y2+
b3+
y3+
prec
b4+
y4+
y5+
b5+
y6+
b6+
KGEDFVK+XL_L
A_b_ion
A_y_ion
intensity
m/z
0
20
40
60
200
300
400
500
600
700
800
y1+
y2+
b2+
b3+
prec
b4+
y4+
y5+
y6+
b6+
TSKDTTASAVAVGLR+XL_S
TSKDTTASAVAVGLR+XL_L
